# Functional illness in primary care: dysfunction versus disease

**DOI:** 10.1186/1471-2296-9-30

**Published:** 2008-05-15

**Authors:** Nefyn Williams, Clare Wilkinson, Nigel Stott, David B Menkes

**Affiliations:** 1 Department of Primary Care and Public Health, Cardiff University, North Wales Clinical School, Wrecsam, UK; 2 Department of Primary Care and Public Health, Cardiff University, UK; 3 Department of Psychological Medicine, Waikato Clinical School, University of Auckland, New Zealand

## Abstract

**Background:**

The Biopsychosocial Model aims to integrate the biological, psychological and social components of illness, but integration is difficult in practice, particularly when patients consult with medically unexplained physical symptoms or functional illness.

**Discussion:**

This Biopsychosocial Model was developed from General Systems Theory, which describes nature as a dynamic order of interacting parts and processes, from molecular to societal. Despite such conceptual progress, the biological, psychological, social and spiritual components of illness are seldom managed as an integrated whole in conventional medical practice. This is because the biomedical model can be easier to use, clinicians often have difficulty relinquishing a disease-centred approach to diagnosis, and either dismiss illness when pathology has been excluded, or explain all undifferentiated illness in terms of psychosocial factors. By contrast, traditional and complementary treatment systems describe reversible functional disturbances, and appear better at integrating the different components of illness. Conventional medicine retains the advantage of scientific method and an expanding evidence base, but needs to more effectively integrate psychosocial factors into assessment and management, notably of 'functional' illness. As an aid to integration, pathology characterised by structural change in tissues and organs is contrasted with dysfunction arising from disordered physiology or psychology that may occur independent of pathological change.

**Summary:**

We propose a classification of illness that includes orthogonal dimensions of pathology and dysfunction to support a broadly based clinical approach to patients; adoption of which may lead to fewer inappropriate investigations and secondary care referrals and greater use of cognitive behavioural techniques, particularly when managing functional illness.

## Background

Our conceptual models of illness shape our consulting behaviour [1]. This paper aims to stimulate debate by developing the biopsychosocial model in primary health care, particularly with regard to undifferentiated illness.

Starfield's definition of primary care [2] as '...first contact, continuous, comprehensive and co-ordinated care provided to populations undifferentiated by gender, disease system or organ system' has achieved international acceptance. Such a broadly defined discipline requires a broad working definition of health or illness, yet such breadth is not always evident in daily practice. A particular challenge to conventional practice is posed by medically unexplained physical symptoms (MUPS), estimated to constitute one in five primary care consultations [3], and a persistent cause of presentation in 2.5% [4]. Despite notional acceptance of the biopsychosocial model, contemporary practice tends to frame such presentations as either psychological or physical, which is compounded by current classification systems [5]. Newer approaches include: assessing, organising and treating these biological, psychological and social components within a four-dimensional grid [6]; addressing unmet psychiatric needs delivered either by psychiatrists in a collaborative care model [7], or by training primary care clinicians in a re-attribution programme [8]. In these interventions the emphasis is shifted from physical to the psychological, but true integration remains elusive. In order to make the biopsychosocial model easier to use in everyday practise, we suggest that illness in primary care be considered in two interacting dimensions:

1. Pathology; characterised by demonstrable structural change in tissues and organs

2. Dysfunction; arising from disordered physiology or psychology, which may occur independent of pathological change

Appropriate care requires that both dimensions are appraised by a skilled clinician, mindful that both may manifest, and interact, in the psychosocial context of the individual patient. Before discussing the implications of this approach for clinical practice, medical education and research, we first consider the development of the biopsychosocial model from general systems theory.

## Discussion

### General Systems Theory

General systems theory offers an intuitively appealing model for understanding illness presenting in primary care. In contrast to determinism, which states that every event has an antecedent cause, nature is seen as a dynamic order of interacting parts and processes [9]. In this hierarchy of systems (e.g., biochemicals to organelle, cell, tissue, organ, organism, family, community, society), there is both vertical and horizontal interaction. For example, the endocrine system interacts with both the nervous and immune systems. Each level has properties not present in its individual parts, but arising from the relationship between the parts. Thus people are more than the sum of their organs and tissues, and society more than the sum of its individuals. If equilibrium is disturbed, reactive forces reverberate in the system until a new equilibrium is established. This model can explain how each level in the hierarchy interacts with increasing complexity, and forms a continuum from the sub-cellular level to the roles and relationships constituting society [10]. It even works to promote concepts of global connectedness [11]. Additional explanatory detail is needed at the level of the individual human being, arguably the level of greatest complexity gain due to sentience and self-concept [12,13].

### The biopsychosocial model

General systems theory provides a structure for systematic thinking about a complex world, and has been used in health care to describe how the physical, psychological and social components of illness can be considered together in primary care [10,14-16], and other branches of medicine [17]. The resulting biopsychosocial model attempts to integrate these three components of illness, and can be distinguished from the biomedical model, where psychosocial aspects are considered separately, if at all [18,19]. A biopsychosocial approach requires broad definitions of health [20,21], provides a framework to address functional illness consisting of MUPS and functional somatic syndromes, as well as the importance of life events and spiritual or existential aspects of care.

#### Medically Unexplained Physical Symptoms (MUPS)

GPs are often presented with illness which defies biomedical understanding and thus differentiation because it is self-limiting; distorted by early treatment or changed circumstance; intermediate between existing labels; a mild or atypical variant of known pathology; not yet understood; or some combination of these [22-24]. Diary studies reveal that individuals tend to develop a new physical symptom on average every five to seven days; the vast majority of which do not result in a consultation. Those consulting doctors are more likely to have had a recent stressful life event [22]. Similarly, individuals with high levels of subjective distress are more likely to notice and complain about internal bodily sensations [25,26]. Psychological distress is linked to both the number and severity of unexplained symptoms [27] and health care use [28]. MUPS are common and are often associated with psychological morbidity, but most patients presenting with them do not have definite psychiatric illness [29,30].

#### Functional somatic syndromes

Clusters of unexplained symptoms are often grouped into syndromes and given 'diagnostic' labels such as irritable bowel syndrome, fibromyalgia or chronic fatigue syndrome. They have been classified according to the secondary care division of specialties and sub-specialties. Thus gastroenterologists label irritable bowel syndrome; cardiologists non-cardiac chest pain; urologists urethral syndrome etc. Many syndrome labels correspond to and can be seen to justify specialists' favourite treatments. Thus non-specific back pain may be described as 'facet syndrome' by anaesthetists who inject joints, 'somatic dysfunction' by osteopaths who treat spinal areas by manipulation, 'instability' by spinal surgeons who fuse spinal vertebrae, 'somatisation' by psychiatrists and 'illness behaviour' by psychologists. Many of these syndromes have common core clinical features [22,31], and often co-exist [32,33], so it is uncertain how much they are distinct clinical entities, or variations on a theme of general body distress [33-36]. As with MUPS, these syndromes tend to have a strong psychological component. A stepped care approach has been advocated using: non-pharmacological treatments such as exercise and cognitive behavioural therapy, which are more effective than passive treatments such as injections and operations, and pharmacological treatments targeted on the central nervous system such as tricyclic antidepressants [37].

#### Psychosocial factors

Psychosocial factors are also important in disease of known pathology. Social isolation, unemployment and other stressful life events are independently associated with higher mortality from all causes [38]. Depression predicts reduced survival following myocardial infarction [39,40], possibly due to an association with fatigue [41] or non-compliance [42], although other studies have failed to replicate this finding [43]. The well-established link between poverty and ill health cannot be fully explained by health behaviours or specific aetiological factors [44,45]. In a more positive sense, social capital has a pervasive beneficial effect on quality of life, measures of physical health, and resilience to stress [46]. Psychological factors, notably stress, are known to affect the neuroendocrine and immune systems [47] and have been linked to cancer and HIV progression [48,49]. Similarly, psychological disorder appears to enhance the development of inflammation and infection in atherosclerotic plaques [50] and activate autoimmune disease such as rheumatoid arthritis [51]. Neuroendocrine and immune effects of extreme stress or depression may be better understood as normal-range responses to the human condition, which in turn have deleterious effects on physical health.

#### Social context

One of the most important functions of medicine through the ages has been as a source of refuge for the sick. This includes hospital admission for healing, relief or protection; official sanction of the sick role in terms of certification or sick pay; refuge from distressing symptoms and the fear of serious disease by the provision of reassurance, symptom relief, and sometimes cure [52].

#### Spiritual dimension

The biopsychosocial model also has an interpretative function, in that it can be used as a source of meaning to individuals' experience of illness [53]. The need to find deeper meaning to life, illness and death is an ancient characteristic of *Homo sapiens*, and the abstract thought involved in this process appears unique to our species. Closely aligned to the phenomenon is an instinct for spiritual practice and religious worship. Spiritual or existential discomfort is rarely formally considered by doctors, except in palliative care [54], or dynamic psychotherapy. Spiritual needs have been defined by one author [55] as "the needs and expectations that all humans have to find meaning, purpose, and value in life". These belief systems may or may not be part of religious faith, but 'spiritual care' is about helping people whose sense of meaning and purpose is challenged by their experience of illness [55].

### Difficulties using the biosychosocial model

#### The biomedical model can be easier to use

Although integrating biological, psychological and social components of illness can accommodate the complexity of MUPS and functional syndromes, it is frequently misunderstood or inadequately applied in clinical practice. It is often viewed as abstract and impractical, perhaps because there is misleading simplicity in the key components of the model.

In routine clinical care, the biological, psychological and social components of illness may be interpreted and managed separately rather than in an integrated manner. Significant symptoms of anxiety or depression are present in 25–52% of primary care patients [56,57], but many go unrecognised because over half present with physical rather than psychological symptoms [58-60], and genuine integration of treatments aimed at both soma and psyche is the exception rather than the rule. This is partly due to time constraints [61], and because clinicians are creatures of habit, using rules of thumb or heuristics to guide their assessments or management decisions, which in turn are heavily influenced by their medical training, methods of classification [62] and clinical experience [63]. Unsurprisingly, many doctors find dualistic practice easier and less stressful. The biomedical model, in which *"disease can be viewed independently from the person who is suffering from it, and from his or her social context" *[10] remains deeply entrenched in contemporary medical practice and teaching.

#### Difficulty letting go of the disease-centred approach to diagnosis

There are many examples of unexplained symptoms given spurious pathological labels. This can be on clinical grounds, such as 'vertebrobasilar insufficiency' for dizziness on neck extension, or 'sciatica' for any pain radiating down the leg. It can arise from investigations revealing minor degrees of abnormality, such as mild gastritis on endoscopy accounting for epigastric pain, marginally abnormal biochemistry explaining lethargy, or mild hypertension as a cause of headache. Moreover, investigations can reveal supposedly pathological changes prevalent in large numbers of the asymptomatic general population, such as cervical and lumbar spondylosis on plain radiographs [64], or intervertebral disc abnormalities on magnetic resonance imaging (MRI) [65].

Over-enthusiastic pathological diagnoses can be damaging to patients in a number of ways, especially when patients collude with or even drive the process. Patients may be over-investigated, referred for unnecessary opinions and procedures, which may have serious adverse consequences[66] and waste resources [67]. Pathological labelling can increase psychological morbidity and the likelihood of somatic fixation [68]. Abnormal ideas about the aetiology of symptoms can lead to unnecessary surgery [69] and inappropriate self-management. For example, as a consequence of being told from radiographic findings that musculoskeletal symptoms are due to 'wear and tear', patients commonly avoid beneficial physical activity. Similarly, anxiety symptoms attributed to a stressful stimulus often lead to avoidance, the exact opposite of what behavioural therapy has demonstrated to be helpful.

#### Defining illness too narrowly in pharmacological terms

There may be a temptation in practice to explain illness entirely in pharmacological and biochemical terms. For example, sumatriptan is useful in acute migraine by interacting with serotonin receptors, so discussion of migraine aetiology and management may be restricted to the serotonin system. A similarly restricted view on the aetiology of depression and schizophrenia results from focusing on the mode of action of antidepressants and antipsychotics on monoamine neurotransmitters [70].

#### Dismissing illness when pathology has been excluded

Non-pathological conditions can be just as disabling as pathologies when generic outcome measures are compared across different conditions [71,72]. Despite this, there is a tendency for medical practitioners, after excluding important pathological diagnoses, to discount patients' symptoms and lose interest in their care. Qualitative studies report that doctors may resort to 'victim blaming' after pathology is excluded [73]. These reactions not only damage the doctor-patient relationship, but encourage abnormal illness behaviour, notably including the quest for 'legitimate' symptoms and investigations, thereby worsening outcomes [74,75].

#### Explaining all undifferentiated illness in terms of psychosocial factors

When no pathological cause can be found for unexplained symptoms, it is tempting to conclude that 'it is all in the mind', especially when there is a strong link with psychological distress. The success of cognitive behavioural therapy, psychiatric consultation within primary care [7] and re-attribution [8] to treat unexplained symptoms has led many to presume that psychological and social factors are paramount, but psychological therapies may be rehabilitative in some patients irrespective of the primary cause of their symptoms. There is also evidence of disturbed physiology in many cases of unexplained symptoms such as fatigue [76]. Moreover, the possibility of undifferentiated illness reflecting a missed physical diagnosis should always be considered [77]. Evidence that psychological therapies may affect brain function in a manner similar to that caused by drug treatments suggests common pathways of symptom genesis and relief [78], and reinforces hypotheses of functional integration of physiology and psychology.

#### Classification systems for functional illness

Mental disorders are very prevalent in primary care and commonly occur together. Functional illness often exists without other mental illness [30]. Traditional classification systems developed in specialised secondary care settings concentrate on its extreme and chronic manifestations. They tend to either classify it with psychiatric disorders or as separate syndromes based on pre-defined checklists, prolonged symptom duration and after organ pathology has been excluded [63]. Newer classification systems are more useful for primary care in that they are split from psychiatry and milder and shorter duration of MUPS are included in a separate category [5]. However even this new classification does not aid integration and presumes that functional illness is a separate entity.

#### Homo Sapiens is body, mind and spirit

As discussed earlier, a narrowly defined clinical role can be easier and less stressful for doctors. Integrating the biological, psychological and social components of healing is difficult enough without the added time and complexity of considering a relevant spiritual or existential dimension. What insights are available from other medical systems to guide us?

### Insights from traditional and complementary medical systems

#### Reversible functional disturbance

Nineteenth century physicians Beard and Dubois [79] described an integrated view of unexplained symptoms and syndromes, and saw them as manifestations of reversible functional disturbance. A non-judgemental, supportive, empathic approach was advocated, which used the strength of the doctor-patient relationship to provide reassurance that symptoms were likely to improve, and to encourage patient autonomy. There has been a long tradition of such a patient-centred approach in central Europe [80], advocated more recently in English-speaking countries [79]. The extended re-attribution and management model is an example of a patient-centred integrative approach used in Danish primary care [8].

#### Eastern medical traditions

Traditional Chinese medicine uses a highly integrated model of illness. Instead of diagnosing disease, traditional Chinese medical practitioners describe a 'pattern of disharmony' or an imbalance in the patient's body. A symptom is not traced back to its source, but is interpreted in terms of the patient's entire bodily pattern. A person who is well or 'in harmony' has no distressing symptoms and is in physical, mental and spiritual balance; in illness, the symptom is only one part of a complete bodily imbalance that can be seen in other aspects of the individual's life and behaviour [81]. Similar principles of maintaining an energetic equilibrium are involved in Ayurvedic medicine [82].

#### Somatic dysfunction

Osteopathy also has an integrated model of illness, based on the concept that symptoms can arise from abnormal functioning of the musculoskeletal system, not dependent on structural pathological processes [83]. Pathology can be defined by its location, structural change, and how this disturbs physiological processes; dysfunction, by contrast, is the result of the interplay of different structures in various locations. The diagnostic task in pathological diagnosis is to localise the lesion exactly, and to determine its nature; in dysfunction to determine the chain of abnormal physiology and psychology, and to assess the importance of the individual links which may be useful in explanation or management. Pathological disease alters the anatomy, sometimes microscopic, of affected tissue; whereas dysfunction in the absence of pathology is a disorder of physiology or psychology. By analogy with computers, pathology is a problem with hardware; dysfunction with software. Modern technology enables clinicians to diagnose pathological conditions more effectively, and with more objectivity. In dysfunction technology is of limited use, integrative clinical skills and effective communication are decisive [84].

### Contrasting pathology with dysfunction

#### Dysfunction in any body system

We propose that the concept of dysfunction can be expanded to include functional syndromes in all of the body's systems, including the brain. Most psychological disturbance is a variant, or an extreme version of commonly experienced cognitions, emotions or behaviours. There is a continuum with normality. In primary mood and anxiety disorders, no structural pathological changes in the brain have been found, although abnormalities of neurotransmitter function have been postulated [85] and are consistent with the observed effects of some treatments [86]. In common mental disorders, including mood, substance use and personality disorders, it is impossible to localise the problem to any single brain structure; the disorders, or at least their manifestations, are often reversible. In contrast, pathological disease brings about irreversible structural change in the brain's morphology, such as in chronic organic brain syndromes (including dementia caused by stroke, demyelination or Alzheimer-type degeneration) and probably also schizophrenia [87]. This distinction between pathology and dysfunction is not completely clear cut, because in some cases dysfunction appears to progress to structural pathology. There is evidence, for example, that untreated depression or seizures may lead to irreversible brain changes [88,89].

#### Pathology and dysfunction as separate dimensions

**Figure 1:**
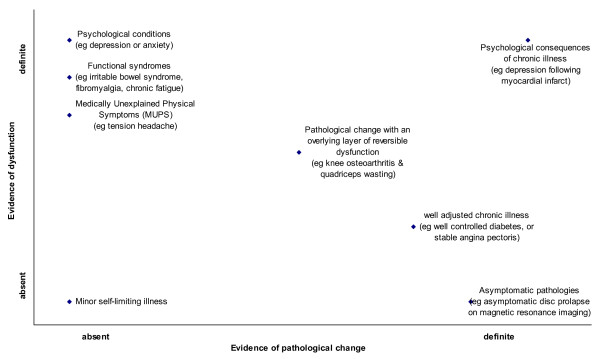
**Hypothetical scatter plot of dysfunction versus pathology in primary care consultations**. **Dysfunction**. abnormal functioning, either physiological or psychological, which is reversible and not dependent on pathological processes. **Pathology**: abnormal functioning caused by structural pathological change, either gross or microscopic.

Evidence for links between body and mind continues to accumulate, vitiating the previously predominant Cartesian dualistic model [22]. One consequence is the biopsychosocial model described above. We propose a further extension for the classification of unexplained symptoms and functional syndromes. Although it seems intuitive that illnesses may arise predominantly from the psyche or the soma, functional illness initiation and progression typically involves both domains. Our proposed classification thus includes orthogonal dimensions of pathology and dysfunction (Figure 1). For this purpose pathology is narrowly defined as pathological processes that cause gross or microscopic structural change in any of the body's tissues, including the brain, resulting in abnormality of function. Dysfunction, by contrast, is abnormal functioning of the body, caused by, or manifested as disturbed physiological or psychological processes independent of known structural pathology.

### Implications for clinical practice, audit, and research

We conclude that routinely contrasting dysfunction with disease facilitates the routine use of the biopsychosocial model in everyday clinical assessment and management, and has implications for teaching and research. Adoption in primary care should be marked by changes in consulting behaviour, some of which would be readily discernible by clinical audit, such as reductions in pathological labelling, fewer investigations and secondary care referrals. Adoption might lead to more explanation of physiological and psychological mechanisms [90,91], and greater use of non-pharmacological interventions such as cognitive behavioural therapy, graded exercise and counselling [37]. Some of these treatments may need to be provided outside conventional medical practice, but patient confidence in the therapeutic plan will be an essential ingredient, particularly if clinicians follow the recently published National Institute for Clinical Excellence (NICE) guideline on chronic fatigue syndrome, where unprecedented emphasis has been placed on the importance of management plans that are understood and approved of by the patient and their carers [92].

## Summary

The biological, psychological and social components of illness are seldom managed as an integrated whole in conventional medical practice. Traditional and complementary medical systems appear better at integrating these three components. As an aid to integration, pathology characterised by structural change in tissues and organs is contrasted with dysfunction arising from disordered physiology or psychology.

## Abbreviations

MUPS: Medically Unexplained Physical Symptoms; HIV: Human Immunodeficiency Virus; MRI: Magnetic Resonance Imaging; NICE: National Institute of Clinical Excellence.

## Competing interests

The authors declare that they have no competing interests.

## Authors' contributions

This debate paper arose from a series of discussions between NW and CW concerning the nature of dysfunction in complementary medicine and its relevance to primary care, particularly in relation to medically unexplained symptoms and psychological disturbance. NW wrote an initial draft which was amended by CW and then passed on to NS and DM who made substantial contributions and changes.

## Pre-publication history

The pre-publication history for this paper can be accessed here:


